# A TRPV Channel Modulates *C. elegans* Neurosecretion, Larval Starvation Survival, and Adult Lifespan

**DOI:** 10.1371/journal.pgen.1000213

**Published:** 2008-10-10

**Authors:** Brian H. Lee, Kaveh Ashrafi

**Affiliations:** 1 Department of Physiology, University of California San Francisco, San Francisco, California, United States of America; 2 UCSF Diabetes Center, University of California San Francisco, San Francisco, California, United States of America; Huntsman Cancer Institute, United States of America

## Abstract

For most organisms, food is only intermittently available; therefore, molecular mechanisms that couple sensation of nutrient availability to growth and development are critical for survival. These mechanisms, however, remain poorly defined. In the absence of nutrients, newly hatched first larval (L1) stage *Caenorhabditis elegans* halt development and survive in this state for several weeks. We isolated mutations in *unc-31*, encoding a calcium-activated regulator of neural dense-core vesicle release, which conferred enhanced starvation survival. This extended survival was reminiscent of that seen in *daf-2* insulin-signaling deficient mutants and was ultimately dependent on *daf-16*, which encodes a FOXO transcription factor whose activity is inhibited by insulin signaling. While insulin signaling modulates metabolism, adult lifespan, and dauer formation, insulin-independent mechanisms that also regulate these processes did not promote starvation survival, indicating that regulation of starvation survival is a distinct program. Cell-specific rescue experiments identified a small subset of primary sensory neurons where *unc-31* reconstitution modulated starvation survival, suggesting that these neurons mediate perception of food availability. We found that OCR-2, a transient receptor potential vanilloid (TRPV) channel that localizes to the cilia of this subset of neurons, regulates peptide-hormone secretion and L1 starvation survival. Moreover, inactivation of *ocr-2* caused a significant extension in adult lifespan. These findings indicate that TRPV channels, which mediate sensation of diverse noxious, thermal, osmotic, and mechanical stimuli, couple nutrient availability to larval starvation survival and adult lifespan through modulation of neural dense-core vesicle secretion.

## Author Summary

Starvation is a common physiological condition encountered by most organisms in their natural environments. However, the molecular mechanisms that allow organisms to accurately sense nutrient availability and match their energetic demands accordingly are not well understood. To elucidate these mechanisms, we isolated mutants in *C. elegans* that survive about 50% longer than wild-type animals when starved. For one such mutant, we found that the extended survival was due to mutation in the *unc-31* gene, which functions in the nervous system to mediate release of neuroendocrine signaling molecules including insulin. Although this gene is broadly expressed in the nervous system, we found that its activity is required in a small subset of sensory neurons to regulate starvation survival. These neurons have ciliated endings that function in detection of environmental cues. Disruption of these cilia, or inactivation of a TRPV channel localized to these cilia, mimicked the perception of nutrient deprivation leading to extended starvation survival, which is dependent on an insulin-regulated transcription factor. Disruption of this channel also extended adult lifespan. Taken together, our findings reveal that TRPV channels couple nutritional cues to neuroendocrine secretion, which in turn determines adult lifespan and larval starvation survival.

## Introduction

In their natural environments, most animals are faced with fluctuating food availability. To survive in this changing environment, animals must be able to coordinate their energy demanding processes such as basal cellular functions, growth, reproduction, and physical activity to available energetic resources. Therefore, the ability to appropriately gauge food availability to initiate programs of growth or arrest is of considerable survival value particularly during starvation. In multicellular organisms, cell-autonomous nutrient sensing mechanisms as well as hormonal cues are thought to ensure coordinated responses among various tissues [1],[2]. However, the molecular identities of nutrient sensors and how they regulate hormonal pathways are not well understood.

*C. elegans* provides a genetically tractable system for uncovering molecular mechanisms of nutrient sensation and starvation resistance. The ability to withstand nutrient deprivation is critical for *C. elegans* survival, as this organism is often found in a starved state in its natural environments [3]. Moreover, *C. elegans* postembryonic growth and development are tightly linked to nutrient availability. *C. elegans* embryos are packaged with sufficient nutrients to support development into the mid-first larval stage. When hatched in favorable conditions, *C. elegans* hermaphrodites undergo four larval transitions (L1–L4) before becoming egg-laying adults. In the absence of food, newly hatched L1 animals arrest development and remain in this diapause state until nutrient is available. L1s in diapause are morphologically similar to well-fed siblings at the same stage. This is in contrast to animals in the hibernating dauer stage, an alternative developmental state characterized by extensive morphological rearrangements (reviewed in [4]). Thus, analysis of L1 diapause provides an opportunity to elucidate starvation survival mechanisms independent of concomitant developmental programs.

As in dauer formation, reduced signaling through the DAF-2/insulin-like receptor is required for maintenance of L1 diapause [5],[6]. Diminished signaling through DAF-2 relieves inhibition of the DAF-16/FOXO transcription factor, which in turn activates transcription of a number of target genes including the *cki-1*/Cip/Kip cyclin-dependent kinase inhibitors [5]. This prevents cell divisions in somatic tissues [5]. Concomitant cell-cycle arrest of the germ-line is independent of *daf-16* and *cki-1* genes but requires other components of the insulin-signaling pathway [6]. Sensory mechanisms that couple diminished nutrient availability to reduced insulin secretion, as well as the cellular sources of insulin signaling that determine reproductive growth or diapause arrest, are unknown.

To identify starvation resistance mechanisms of L1s in diapause, we screened for mutants with enhanced capability to survive starvation. We isolated loss-of-function mutations in *unc-31*, a regulator of dense-core vesicle release that extended starvation survival. Extended survival of *unc-31* mutants was dependent on the activity of insulin- regulated DAF-16/FOXO transcription factor. Cell-specific reconstitution of *unc-31* function led to identification of a small subset of ciliated sensory neurons that regulate starvation survival. We found that inactivation of *ocr-2*, encoding a transient receptor potential vanilloid (TRPV) channel that localizes to these sensory cilia, also extended larval starvation survival as well as conferring extended adult lifespan in a DAF-16/FOXO dependent manner. These findings demonstrate that the decision to grow or remain in diapause is under neural control and reveal a previously unidentified role for the polymodal TRPV channels as ancient sensory gauges that couple nutrient availability to larval starvation survival and adult lifespan.

## Results

### Loss of *unc-31* Extends L1 Starvation Survival

**Figure 1:**
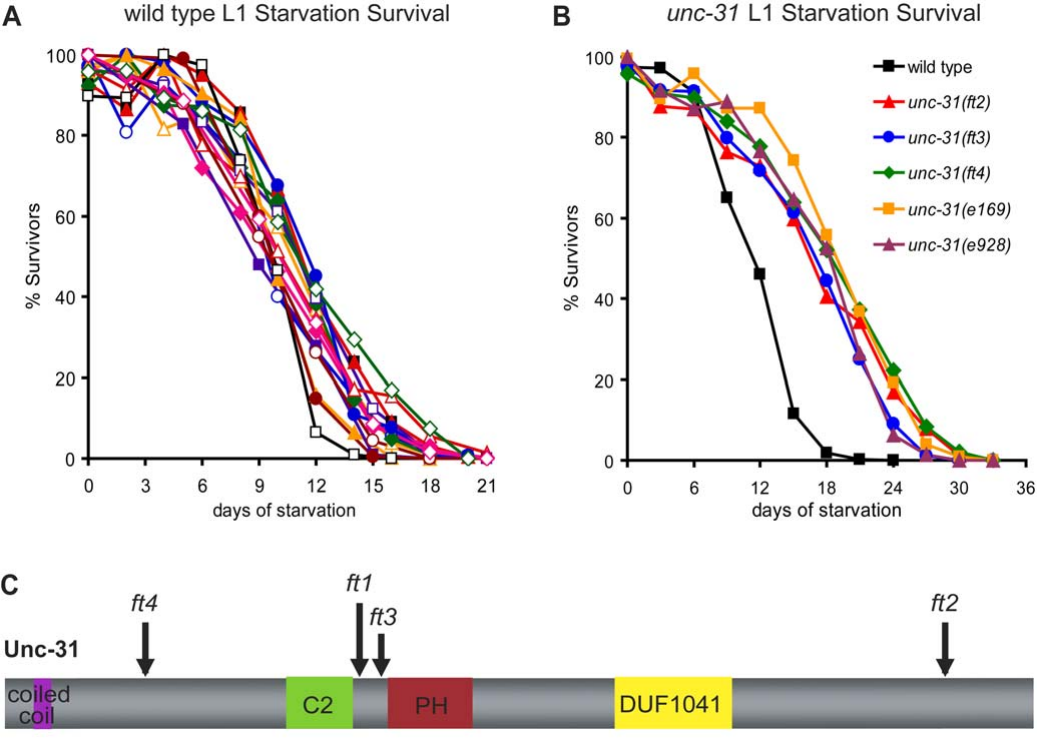
Loss of *unc-31* enhances L1 starvation survival. (A) Multiple independent experiments at room temperature (∼23°C) showing the range and reproducibility of L1 starvation survival distribution for wild-type (N2) animals. Survival curves represent counts on 1000–3000 animals per experiment. In aggregate, average mean survival for wild type was 12.2 days (standard error: ±0.4 days). (B) Multiple *unc-31* loss-of-function mutations extend L1 starvation survival by ∼50%. This difference is statistically significant as determined by log-rank test (p,<0.00001); see Table S1 for details. (C) Predicted protein structure of UNC-31 with functional domains. The locations of the four new *unc-31* mutations, *ft1–ft4*, are indicated. *ft1* causes a W_597_ TGG->TGA stop mutation. *ft2* causes a 2 bp deletion after Q_1304_ resulting in a frame-shift that produced a stop codon four amino acids after the deletion. *ft3* causes a L_610_ TTA->TAA stop mutation. *ft4* causes a R_296_ CGA->TGA stop mutation. Amino acid positions are numbered based upon Wormbase Release WS189 predicted protein structure of UNC-31. C2 = Ca^2+^ binding domain. PH = pleckstrin homology domain. DUF1041 = domain of unknown function likely to be involved in vesicle secretion.

To identify regulators of larval starvation survival, we developed an assay to measure survival of L1 worms under nutrient deprived conditions (see Materials and Methods). We defined starvation survival as the ability to resume growth upon reintroduction to food. Subjecting a population of L1s to starvation resulted in a reproducible distribution of survival rates (Figure 1A). This distribution was dependent upon assay conditions, particularly temperature. For instance, the mean survival rate of animals was increased from ∼12 days at 23°C to ∼17 days at 20°C (compare Figures 1B with 2A for wild type). Therefore, for each set of comparisons, all relevant genotypes were examined as part of the same experiment.

**Figure 2 pgen-1000213-g002:**
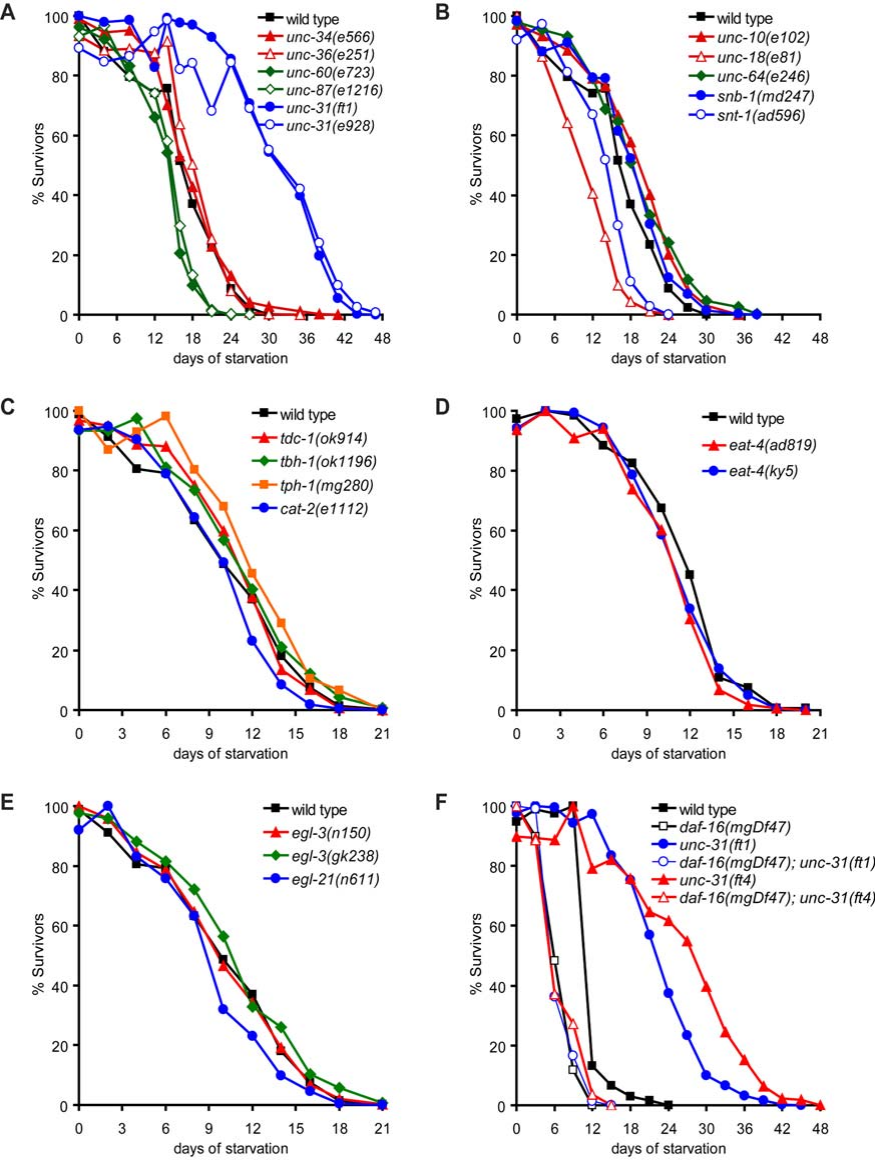
Extended survival of *unc-31* mutants depends on *daf-16*. Effects of mutations that cause (A) paralysis, (B) defects in synaptic vesicle release, (C) defects in synthesis of biogenic amines, (*tph-1*: serotonin, *tdc-1*: tyramine and octopamine, *tbh-1*: octopamine, and *cat-2*: dopamine) (D) defects in glutamate signaling, (E) defects in neuropeptide processing, and (F) loss of DAF-16/FOXO transcription factor, on L1 starvation survival. Statistical analyses of data shown in (A–F) are reported in Table S1. Assays reported in (A–B) were conducted at 20°C resulting in proportional extensions in mean and maximal survival of all strains.

To identify genes that regulate starvation survival, we conducted an EMS (ethyl methanesulfonate) mutagenesis screen for mutants with enhanced starvation survival and recovered twenty-eight mutant lines. At least 50% of starving L1s from each of the mutant lines could resume growth upon reintroduction to food at a time when all starving wild-type controls had lost this capacity (data not shown). Of these twenty-eight lines, ten were severely uncoordinated and this Unc phenotype was linked to increased survival. The Unc phenotype was mapped to the *unc-31* locus using standard single nucleotide polymorphism mapping techniques [7]. Sequencing these ten Unc mutants identified four new alleles (*ft1*–*ft4*) of *unc-31*, with *ft1*, *ft3* and *ft4* likely to be null alleles (Figure 1C). In over 30 independent experiments, loss of *unc-31* resulted in a ∼50% increase in both mean and maximal survival relative to wild type (Figures 1–5 and Table S1). Although maintenance of animals at 20°C already resulted in an extension of mean and maximal survival of all genotypes, loss of *unc-31* still conferred a greater than 50% extension compared to wild type at that temperature (Figure 2A). Previously identified *unc-31* alleles, *e169* and *e928*, also showed enhanced starvation survival similar in extent to the newly identified alleles (Figure 1B, 2A, Table S1). Additionally, expression of an *unc-31* cDNA in neurons fully rescued the extended survival phenotype of *unc-31(ft1)* mutants (Figure 4A). Together, these data indicated that loss of *unc-31* caused an extension in L1 starvation survival.

**Figure 3 pgen-1000213-g003:**
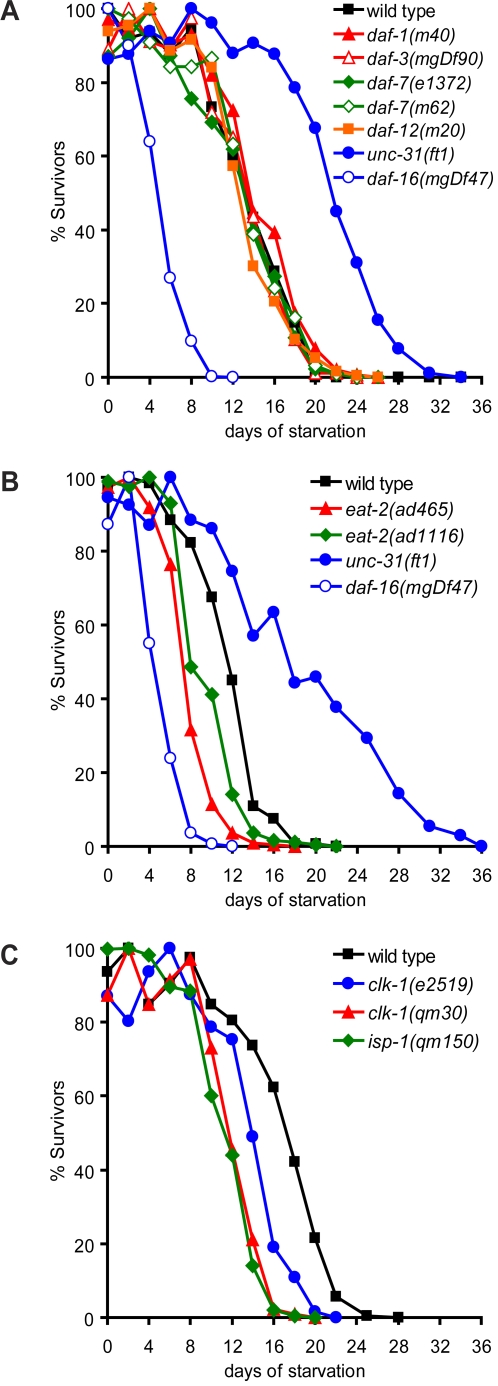
Insulin-independent dauer and longevity mutants do not enhance L1 starvation survival. (A) Effects of mutations in TGF-β signaling pathway on starvation survival. (B–C) Effects of insulin-independent adult longevity mutants on starvation survival. In the case of *clk-1* and *isp-1*, the starvation assays were conducted at 20°C. Statistical analyses of data shown in (A–C) are reported in Table S1.

**Figure 4 pgen-1000213-g004:**
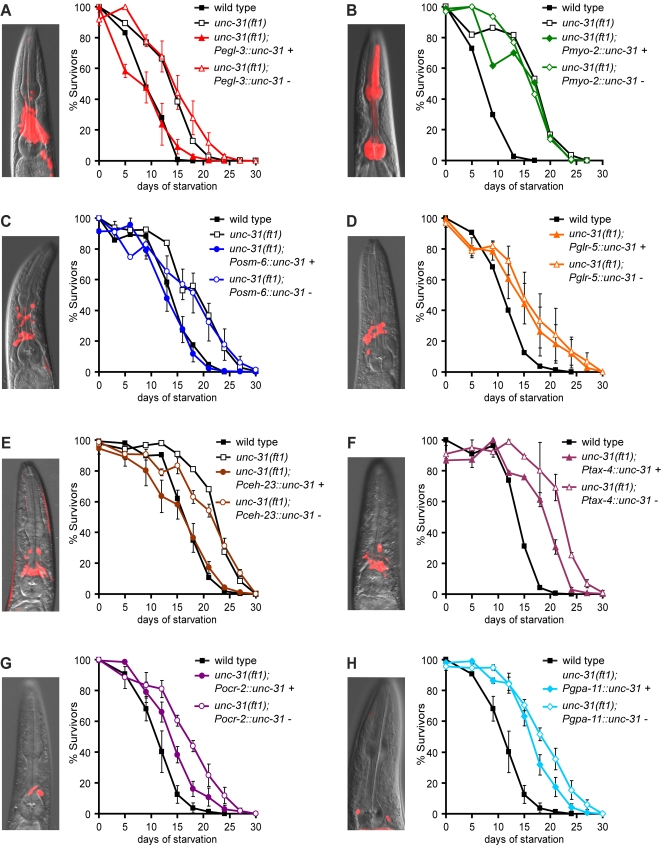
Expression of *unc-31* in ADL and ASH sensory neurons is sufficient to partially abrogate extended starvation survival. (A–H) *unc-31* cDNA separated from a *mCherry* reporter by an intercistronic region was expressed in various tissues of *unc-31(ft1)* mutants and starvation survivals of transgenic animals and non-transgenic siblings were assayed. The mCherry reporter allowed for verification of expression patterns ascribed to each promoter. Examples of the expression pattern of each promoter are shown. For each transgenic line, “+” designates transgenic animals and “−” designates non-transgenic siblings. Expression of *unc-31* using (A) a pan-neuronal *egl-3* promoter, and (C) a ciliated sensory neuron *osm-6* promoter, fully abrogate *unc-31(ft1)* extended starvation survival, while expression using (B) *myo-2*, a pharyngeal muscle promoter, and (D) *glr-5*, an interneuron promoter, do not alter extended starvation survival. (E–H) Reconstitution of wild-type *unc-31* in *unc-31(ft1)* mutants with promoters that target various subsets of ciliated sensory neurons. Individual neurons targeted by each promoter are listed in Table S2. Graphs (except for the *myo-2* negative control) depict averages of 2–4 independent transgenic lines and their non-transgenic siblings along with standard error of the mean for each time point.

**Figure 5:**
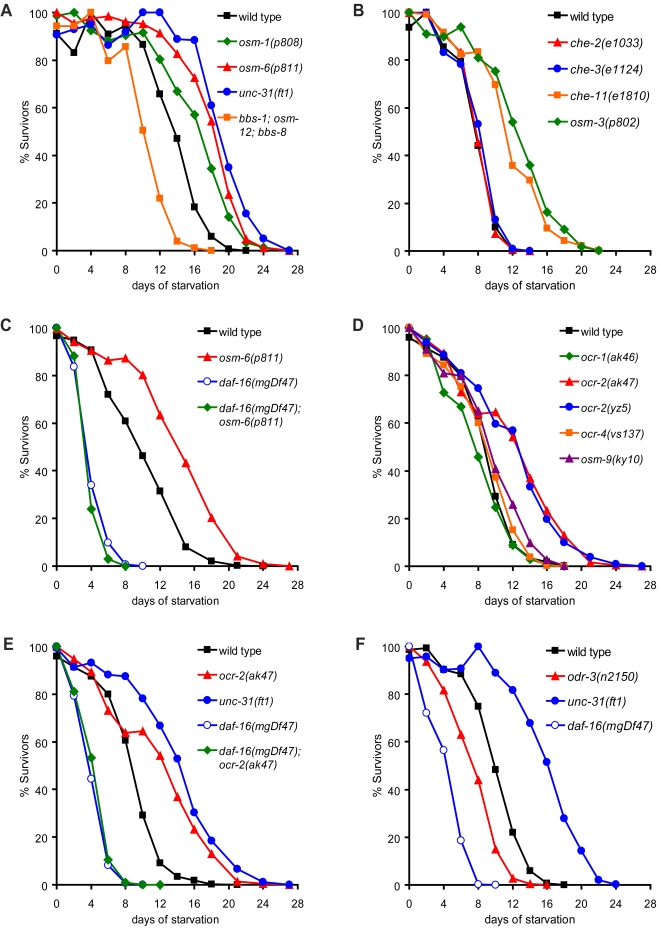
Defective cilia function extends L1 starvation survival. (A–B) Effects of ciliary defects on starvation survival. Only mutations that cause severe defects in cilia function enhance starvation survival. (C) Extended L1 starvation survival of cilia defective *osm-6* mutants requires *daf-16*. (D) Effects of mutations in TRPV family members on starvation survival. *ocr-2* mutants extend starvation survival while other TRPV members do not alter survival. (E) Extended survival of *ocr-2* mutant is dependent on *daf-16*. (F) Loss-of-function mutation in G_α_ *odr-3* reduces starvation survival. Statistical analyses of data shown in (A–F) are reported in Table S1.

Finally, while background mutations could potentially contribute to differences in starvation survival, this effect appeared to be minimal. We obtained similar starvation survival curves for mutants that were outcrossed to the Ashrafi lab wild-type strain as those received from other labs without further outcrossing (For example compare *unc-31* mutants in Figure 1B; details of strain background for all strains examined are reported in Table S1).

### Extended Starvation Survival of *unc-31* Mutants Requires *daf-16*

*unc-31* encodes the *C. elegans* homolog of mammalian CAPS (Ca^2+^ activated protein for secretion) required for the regulated release of dense-core vesicles, which contain biogenic amines, neuropeptides, and insulins [8]–[11]. *unc-31* is broadly expressed in the *C. elegans* nervous system, but not in other cell types [12], and mutants in *unc-31* were originally identified based on their dramatically reduced movement [13]. As *unc-31* mutants are pleiotropic, we sought to determine whether reduced movement, general defects in synaptic transmission, or reduced secretion of specific dense-core vesicle cargoes could account for their enhanced starvation survival phenotype.

Animals with mutations in *unc-34*, encoding an axon guidance cue [14], *unc-36*, encoding a voltage-gated Ca^2+^ channel [15], *unc-60*, encoding a regulator of the actin cytoskeleton [16], and *unc-87*, encoding a muscle myofilament component [17], displayed movement defects that were as severe as those of *unc-31* mutants, yet all had either wild-type or reduced L1 starvation survival (Figure 2A, Table S1). Similarly, mutations in *unc-10*/rim-1, *unc-18*, *unc-64*/syntaxin, *snb-1*/synpatobrevin, and *snt-1*/synpatotagmin, encoding various components of neurotransmitter secretion (reviewed in [18]), did not produce dramatic extensions in starvation survival. Although mutations in *unc-10* and *unc-64* caused statistically significant extensions in mean survival, these extensions were marginal compared to the extension seen in *unc-31* mutants (Figure 2B, Table S1). Together, these findings suggested that starvation resistance of *unc-31* mutants is unlikely a consequence of reduced energy expenditure that might accompany diminished movement or solely a consequence of general defects in neural synaptic activity.

To determine whether loss of dense-core vesicle cargoes might confer extended starvation survival, we examined various biogenic amine, neuropeptide, and insulin-signaling mutants. Animals deficient in synthesis of octopamine, tyramine, or dopamine, (reviewed in [19]) had similar rates of L1 starvation survival as wild-type animals while serotonin-deficient *tph-1* mutants displayed a statistically significant but modest increase in mean survival (Figure 2C, Table S1). In addition, glutamate signaling defective, *eat-4* mutants [20] also displayed wild-type starvation survival rate (Figure 2D, Table S1). Similarly, a null mutation in *egl-3*, a convertase, and a loss-of-function mutation in *egl-21*, a carboxypeptidase, required for the processing of most, but not all, *C. elegans* neuropeptides [21]–[23], did not alter starvation survival (Figure 2E, Table S1). Thus, while serotonin, tyramine, octopamine, glutamate, dopamine, and neuropeptide signaling pathways function in a variety of food-related behaviors [24]–[26], defects in these pathways did not appreciably affect long-term starvation survival. A role for neuropeptide signaling in starvation survival, however, could not be definitively ruled out, as *egl-3* and *egl-21* mutants are not required for the processing of all neuropeptides [21]–[23].

Finally, we examined components of the insulin-signaling pathway. Loss of function of the insulin receptor, *daf-2*, was previously shown to increase starvation survival [5]. Phenotypes associated with reduced DAF-2/insulin-receptor signaling such as dauer entry and extended lifespan are suppressed by loss-of-function mutations in the FOXO transcription factor, *daf-16* [27],[28]. Relative to wild-type animals, *daf-16* null mutants had reduced L1 starvation survival and combination of *daf-16(mgDf47)* with either *unc-31(ft1)* or *unc-31(ft4)* completely suppressed the extended survival of these mutants (Figure 2F, Table S1). This indicated that the enhanced L1 starvation survival of *unc-31* mutants was dependent on the activity of the insulin-regulated DAF-16/FOXO transcription factor. These results were consistent with previous reports showing that the extended adult lifespan of *unc-31* mutants was dependent on *daf-16* [29]. Together, these results suggested that the extended survival of *unc-31* mutants is likely due to diminished insulin signaling. These results, however, do not rule out the possibility that *unc-31* may regulate starvation survival through DAF-16 but independent of insulin signaling.

### L1 Starvation Survival Is Distinct from Insulin-Independent Mechanisms of Dauer Formation and Adult Lifespan

Loss of insulin signaling and subsequent activation of DAF-16 during early development causes formation of stress-resistant, long-lived dauers (review in [4]). Therefore, we determined whether other dauer regulatory pathways also affect L1 starvation survival. Similar to loss of *daf-2*, loss of either *daf-7* or *daf-1*, encoding a neurally expressed TGF-β ligand and its receptor, respectively, promote dauer entry (reviewed in [4]). However, unlike *daf-2* [5],[30], mutations in *daf-1* and *daf-7* did not alter L1 starvation survival (Figure 3A, Table S1). Consistent with this observation, loss of *daf-3*, encoding a co-SMAD required for dauer entry when TGF-β signaling is inhibited, or loss of *daf-12*, encoding a nuclear hormone receptor whose activity is critical for dauer entry when either insulin or TGF-β signaling pathways are inactivated (reviewed in [4]), did not affect L1 starvation survival (Figure 3A, Table S1). These findings suggested that distinct mechanisms operate at different stages of development to regulate starvation survival programs.

Reduced insulin signaling also causes extended adult lifespan, therefore, we examined whether other longevity pathways also influence L1 starvation survival. Caloric restriction mutants such as *eat-2*, which are defective in food intake, extend lifespan in a *daf-16*-independent manner [31],[32]. *eat-2* mutants, however, displayed reduced L1 starvation survival (Figure 3B) suggesting that extended starvation survival did not correlate with extended adult lifespan. Similarly, *isp-1* and *clk-1* mitochondrial respiratory mutants, which also display *daf-16*-independent extensions in adult lifespan [33],[34], had diminished L1 starvation survival (Figure 3C). Thus, with the exception of insulin signaling, other pathways that promote adult lifespan had detrimental effects on starvation survival suggesting that mechanisms that regulate adult lifespan can be dissociated from mechanisms that regulate starvation survival.

### *unc-31* Functions in a Subset of Sensory Neurons to Regulate Starvation Survival

To identify cellular sites of *unc-31* function in the modulation of L1 starvation survival, we selectively expressed wild-type *unc-31* cDNA in subsets of sensory, pharyngeal, and interneurons as well as body and pharyngeal muscles of *unc-31(ft1)* mutants (Table S2). As expected from its neuronal-specific expression pattern [12], *unc-31* reconstitution using a pan-neuronal *egl-3* promoter [35] fully abrogated the extended survival of *unc-31(ft1)* animals (Figure 4A, Table S2), whereas expression under the pharyngeal *myo-2* or muscle-specific *myo-3* promoters [36] had no effects (Figure 4B, Table S2). *unc-31* expression in all ciliated sensory neurons using the *osm-6* promoter [37] reverted the extended starvation survival of *unc-31(ft1)* mutant to wild-type levels (Figure 4C, Table S2) whereas expression in many interneurons using the *glr-5* or *glr-2* promoters or in pharyngeal neurons under the *glr-8* promoter [38] did not alter the increased survival rate of this mutant (Figure 4D, Table S2). Together, these findings suggested that UNC-31 function in sensory neurons was sufficient to modulate the extent of L1 starvation survival.

We next targeted subsets of sensory neurons using the *ceh-23* and *tax-4* promoters (Table S2). Reconstitution of *unc-31* using a *ceh-23* promoter [39] fully abrogated the extended starvation survival of *unc-31(ft1)* mutants (Figure 4E, Table S2), while expression using a *tax-4* promoter [40] resulted in an intermediate phenotype (Figure 4F, Table S2). Since the *ceh-23* and *tax-4* promoters target many of the same neurons (Table S2), we considered the possibility that sensory neurons targeted by the *ceh-23*, but not the *tax-4*, promoter (amphid sensory ADL and ASH neurons and tail sensory phasmid neurons) play an important role in determining L1 starvation survival. To examine the role of this subset of neurons, we used an *ocr-2* promoter [41] to target *unc-31* to ADL, ASH, AWA, and ADF head sensory neurons as well as PHA and PHB tail phasmid sensory neurons and a *gpa-11* promoter [42] to only target ADL and ASH neurons. Reconstitution of *unc-31* using either of these promoters was sufficient to partially abrogate extended L1 starvation survival of *unc-31(ft1)* mutants (Figures 4G–H, Table S2). Thus, UNC-31 function in a small number of sensory neurons, particularly ADL and ASH, is sufficient to modulate the extent of L1 starvation survival.

### Inactivation of a Cilia-Localized TRPV Channel Extends L1 Starvation Survival

Expression of *unc-31* in ciliated neurons fully rescued *unc-31(ft1)* mutants, suggesting that defects in sensory perception might regulate starvation survival. Cilia are specialized organelles that are enriched in sensory transduction mechanisms and disruption of cilia cause defects in sensation of environmental cues (reviewed in [43]). We postulated that disruption of cilia-localized mechanisms of food sensation might regulate starvation survival. Indeed, we found that *osm-1(p808)*, *osm-3(p802)*, *osm-6(p811)*, and *che-11(e1810)* mutants, which have shortened and defective sensory cilia [44], displayed extended L1 starvation survival comparable to that of *unc-31* mutants (Figure 5A–5C, Table S1). *osm-1*, *osm-3*, *osm-6*, and *che-11* encode various components of intraflagellar transport machinery required for cilia formation and function (reviewed in [45]). Pronounced cilia defects were required to cause enhanced L1 starvation survival, since *che-2(e1033)*, *che-3(e1124)*, and *bbs-1(k1111)*; *osm-12(n1606)*; *bbs-8(nx77)* triple mutants [46], which have less severe defects in cilia function, had wild-type starvation survival rate (Figure 5A, 5B, Table S1). Similar to *unc-31* mutants, the extended survival of *osm-6* mutants was fully dependent on *daf-16* (Figure 5C, Table S1). Furthermore, *unc-31(ft1)*; *osm-6(p811)* double mutants did further enhance starvation survival, suggesting that defects in cilia function and dense-core vesicle release likely function in the same genetic pathway to regulate starvation survival (data not shown).

We next sought to identify specific ciliary nutrient sensing mechanisms that could extend starvation survival. Given that reconstitution of *unc-31* with an *ocr-2* promoter abrogated extended L1 starvation survival of *unc-31(ft1)* mutants (Figure 4G), we examined whether the OCR-2 channel itself played a role in the regulation of starvation survival. Indeed, both *ocr-2(ak47)* null and *ocr-2(yz5)* hypomorphic mutants displayed a ∼30% enhancement in starvation survival (Figure 5D, Table S1).

*ocr-2* encodes a channel of the transient receptor potential vanilloid subfamily, TRPV, that functions in *C. elegans* olfaction, nociception and osmosensation [41]. OCR-2 localizes to the tips of sensory cilia and is, therefore, anatomically well-positioned to gauge changes in environmental cues [41]. To determine the specificity of *ocr-2* inactivation on extension of L1 starvation survival, we examined mutants in other TRPV channels. The *C. elegans* genome encodes for five members of the TRPV family, *ocr-1* through *ocr-4* and *osm-9* [41]. Null mutations in *osm-9* as well as *ocr-1* and *ocr-4* did not alter L1 starvation survival (Figure 5D, Table S1). Given that *ocr-2* and *osm-9* mutations produce similar defects in a number of sensory modalities [41],[47],[48], our findings pointed to a unique role for *ocr-2* in the regulation of L1 starvation survival. Accordingly, we found that mutations in *odr-3*, encoding a G-protein α subunit that functions upstream of OCR-2/OSM-9 channels in mediating olfactory and osmotic sensation [41], did not enhance L1 starvation survival (Figure 5F, Table S1).

### *ocr-2* Regulates Insulin Release from ADL Chemosensory Neurons

**Figure 6:**
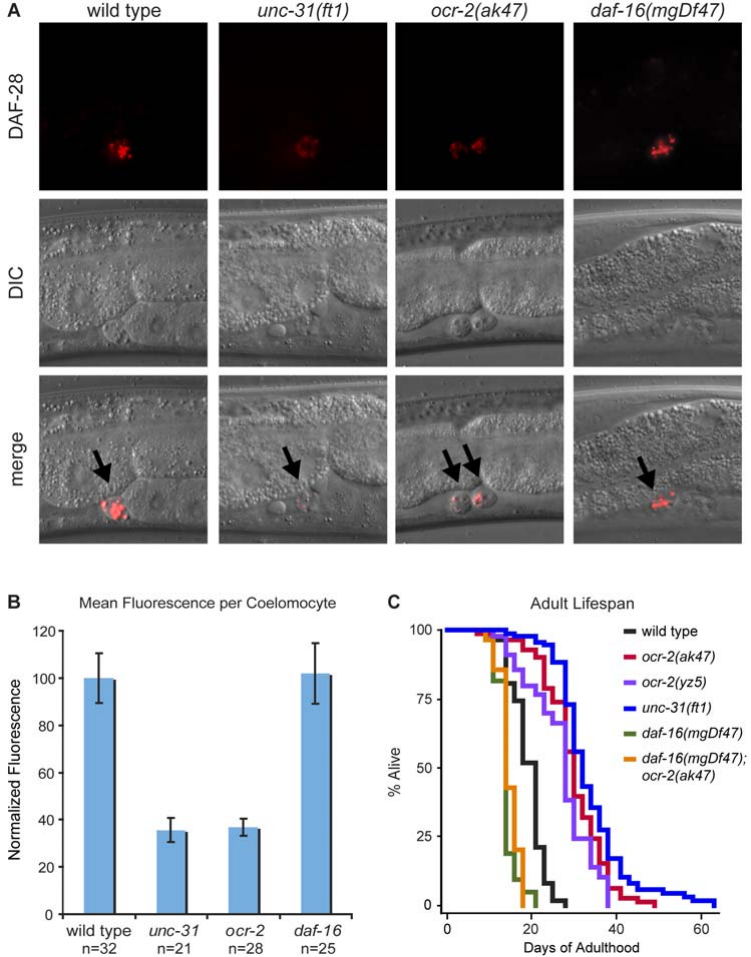
Loss of *ocr-2* reduces neural insulin secretion and increases lifespan. (A–B) Neuronal secretion from ADL neurons was assessed by monitoring the uptake of a fluorescently-tagged insulin, DAF-28::mCherry expressed exclusively in ADL neurons from an *shr-220* promoter, into coelomocytes. Representative images with arrows pointing to coelomocytes (A) and corresponding quantitations normalized to wild type (B) are shown. Bars indicate standard error of the mean. (C) Lifespans of two independent *ocr-2* mutants on plates containing FUDR, a drug that inhibits progeny production. Loss of *ocr-2* substantially extends adult lifespan (p-value<0.0001 as determined by log-rank test; detailed statistical analyses are reported in Table S3). While these two *ocr-2* mutants were generated in two independent laboratories and were not outcrossed to our lab's wild-type strain, they had similarly extended mean and maximal lifespans, suggesting that background differences are unlikely to contribute significantly to their observed lifespan phenotypes.

To determine whether *ocr-2* and *unc-31* mutations extended survival through a common mechanism, we first established that extended survival of *ocr-2(ak47)* mutant was dependent on *daf-16* (Figure 5E, Table S1). We then took advantage of a recently developed assay of insulin uptake by coelomocytes [49] to directly examine whether *unc-31* and *ocr-2* mutations alter insulin secretion from ciliated sensory neurons. Coelomocytes are scavenger cells that take up molecules secreted into the pseudocoelom, a fluid-filled cavity through which secreted hormones gain access to distant tissues. We expressed *daf-28*, encoding an insulin [50], C-terminally fused to *mCherry*, exclusively in ADL chemosensory neurons using an *srh-220* promoter [51]. Expression of this reporter fusion in ADL neurons could be observed in L1-stage animals and throughout subsequent development (data not shown). Moreover, we could detect uptake of the secreted fluorescent insulin by coelomocytes. Loss of *unc-31* and *ocr-2* decreased coelomocyte accumulation of this insulin reporter to ∼35% of wild-type levels (Figure 6A, 6B). Double mutants between *ocr-2* and *unc-31*, however, were not examined as they are closely linked genetic loci. The cilia defective *osm-6* mutant, as expected, reduced accumulation of the insulin reporter to a similar extent as *unc-31* and *ocr-2* mutants (data not shown). Importantly, in the *ocr-2* mutant, coelomocyte uptake of GFP-tagged UNC-122 neuropeptide [52] was unchanged, suggesting that reduced insulin accumulation in coelomocytes was likely due to diminished release from ADL neurons rather than compromised coelomocyte uptake function (data not shown). Additionally, exposure of transgenic L1 animals to starvation caused a reduction in coelomocyte accumulation of DAF-28::mCherry, indicating that this reporter fusion was subjected to nutritional controls (data not shown). Together, these findings supported the notion that the extended L1 starvation survival of *unc-31* and *ocr-2* mutants was associated with decreased neural secretion of dense-core vesicles that contain neuroendocrine signaling molecules such as insulin-like peptides.

### Loss of *ocr-2* Extends Adult LifeSpan

Our findings raised the possibility that *ocr-2* mutants may have diminished insulin signaling. Since insulin-signaling deficient animals have extended adult lifespan, we determined the adult lifespan of *ocr-2* mutants. Relative to wild-type animals, inactivation of *ocr-2* caused a substantial extension in lifespan, and as previously reported [29], *unc-31* mutations also caused extended adult lifespan (Figure 6C, Table S3). As in starvation survival, the extended lifespan of *ocr-2* mutants was dependent on *daf-16* (Figure 6C, Table S3). Unlike *daf-2* mutants, growth rates of wild-type and *ocr-2(ak47)* worms were indistinguishable at room temperature [48]. Together, these finding indicated that *ocr-2* inactivation moderates insulin signaling to a threshold sufficient for bypass of dauer entry, allowing for normal growth and reproduction, yet sufficiently reduced to enhance larval starvation survival and adult lifespan.

## Discussion

**Figure 7:**
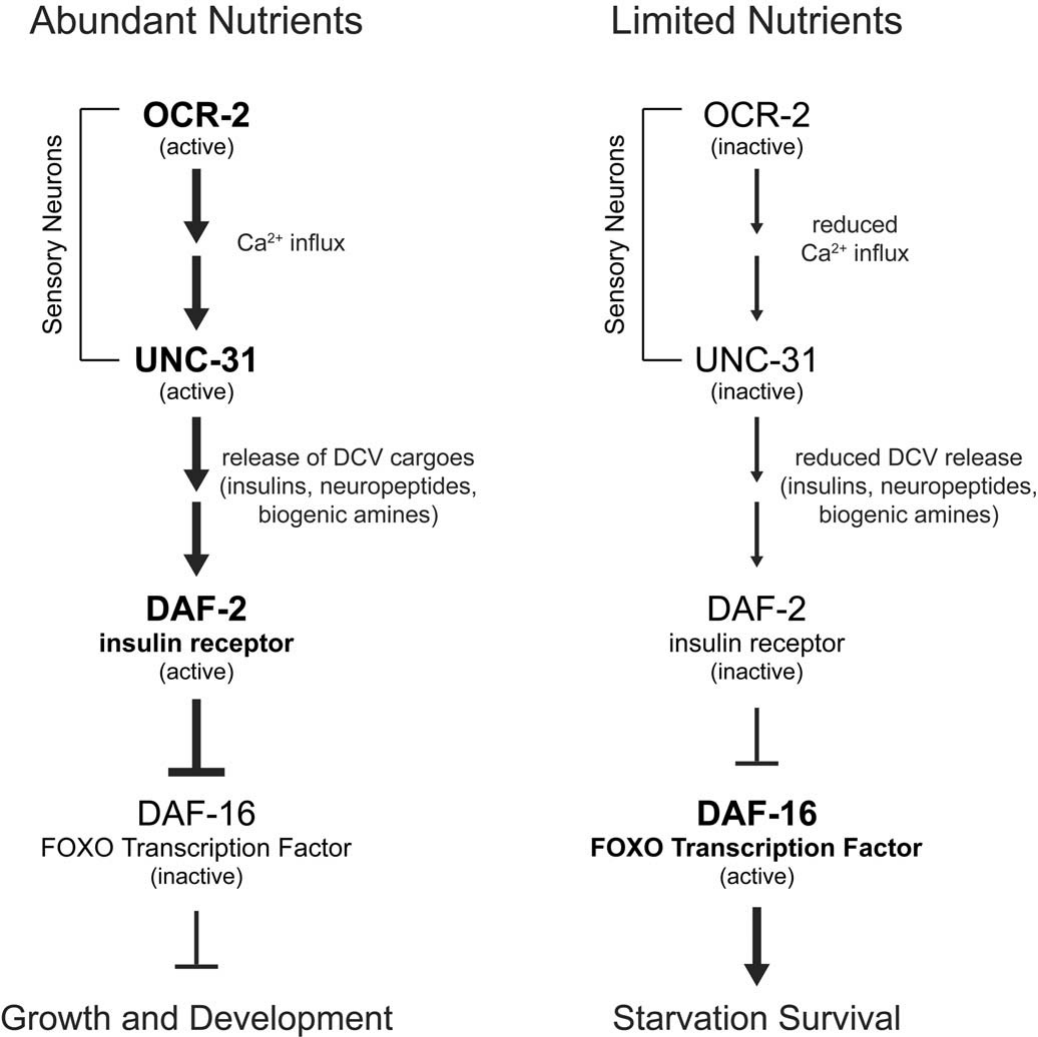
Model of L1 starvation survival regulation by *ocr-2* and *unc-31*. Under favorable environmental conditions, OCR-2, TRPV channel in sensory neurons allows influx of cations such as calcium. In response, calcium-activated UNC-31 promotes release of cargoes contained in dense-core vesicles of sensory neurons. Whether calcium influx from OCR-2 channels directly activates UNC-31 or whether it does so through an intermediate signaling cascade is not yet known. Similarly, the epistatic relationship between *unc-31* and *ocr-2* remains to be established but *ocr-2* is likely to act upstream of *unc-31* based upon their molecular functions. Active release of dense-core vesicle cargoes ultimately inhibits the FOXO transcription factor, DAF-16. This is most likely through direct signaling by insulin-like peptides through the DAF-2 insulin receptor, although alternative mechanisms are possible. Inhibition of DAF-16 favors pathways that promote growth and development. Inhibition of DAF-16 is relieved during unfavorable environmental conditions such as limited nutrient availability, leading to activation of starvation survival mechanisms. Loss of *ocr-2*, *unc-31*, and *daf-2* extend starvation survival either by pre-conditioning animals through expression of starvation resistance genes even during periods of nutrient availability or by preventing spurious inactivation of DAF-16 during starvation.

The ability to sense and adapt to fluctuating environmental conditions and withstand periods of nutrient deprivation carries significant evolutionary advantage. To better understand mechanisms of starvation survival, we conducted a mutagenesis screen and found that mutations in *unc-31* enhance starvation survival. This indicated that diminished neural release of dense-core vesicles enhances starvation resistance. Using functional reconstitution studies, we identified a small subset of ciliated sensory neurons that are likely to directly participate in the perception of nutrient availability and to convey this information to the rest of the organism via regulated secretion of molecules contained in dense-core vesicles. Although these vesicles contain a number of diverse cargoes such as insulins, biogenic amines and neuropeptides, our findings indicated that modulation of insulin-regulated DAF-16/FOXO transcription factor is the principle mechanism that determines the extent of survival during periods of nutrient deprivation. Therefore, the simplest model consistent with these results is that neural assessment of nutrient availability coordinates growth and starvation survival pathways directly through release of insulin-like peptides contained in neural dense-core vesicles (Figure 7). However, definitive proof of this model awaits identification of specific insulins that regulates starvation survival and whose release is modulated by nutritional conditions and UNC-31.

The specific molecular mechanisms through which sensory neurons detect changes in food availability remain to be deciphered; however, our findings suggest a key role for the OCR-2 TRPV channel. This channel is localized to sensory cilia of the specific subset of neurons in which *unc-31* expression was sufficient to modulate starvation survival. Since the TRPV family encodes for nonselective cation channels with a preference for calcium (reviewed in [53]), it is possible that OCR-2 inhibition may directly affect insulin secretion through UNC-31, a Ca^2+^ dependent regulator of dense-core vesicle release [10] (Figure 7). Our data suggest that OCR-2 is only one of several sensory transduction mechanisms that couple nutritional cues to L1 starvation survival, as null mutations in *ocr-2* did not extend survival to the same extent as loss-of-function mutations in *unc-31* and *daf-2* (Figure 5E, Table S1). Thus, multiple sensory mechanisms are likely to converge on dense-core vesicle secretion and insulin signaling to determine starvation survival.

In mammals, TRPV channels respond to noxious, thermal, osmotic, and mechanical stimuli [54]–[56]. Similarly, *C. elegans* TRPV channels are polymodal (reviewed in [57]). OCR-2 functions in ASH neurons to sense noxious nose touch and high osmotic pressure and acts in ADL neurons to avoid repulsive volatile odorants [41]. Consistent with our results, several other reports indicate that the OCR-2 channel also functions in food-related pathways. For instance, this channel is required for social aggregation, a food-related behavior [47], as well as transcriptional expression of the serotonin biosynthetic gene, *tph-1*, in ADF ciliated-sensory neurons [48]. As in mammals, *C. elegans* serotonin is a sophisticated modulator of food-related behaviors [26],[58],[59]. Additionally, a complex mixture of OCR-1, OCR-2, and OCR-4 channels redundantly control egg laying [60], a physiological response that is subject to nutritional regulation (reviewed in [61]). Together, these results indicate that OCR-2 may be a general sensor of nutrient availability that, in turn, modulates a variety of food-related pathways.

*C. elegans* adult lifespan, as in L1 starvation survival, is regulated by sensory perception [62]–[64] and insulin signaling [27],[28]. The *C. elegans* genome encodes for ∼40 insulin-like peptides, many of which display complex overlapping neural expression patterns [65]. With a few notable exceptions [50], [65]–[67], little is known about the identities of specific insulins that regulate various processes attributed to insulin signaling, their cellular sources of secretion, and molecular mechanisms that regulate their release. While the involvement of cilia in the determination of lifespan was previously established [62], our studies raise the possibility that OCR-2 is a key molecular integrator of sensory cues that modulate lifespan through neural insulin signaling. Interestingly, the AWA pair of olfactory neurons are among the few neurons that, when ablated, extend lifespan [63]. However, *ocr-2* is likely to function in other neurons besides AWA as the extension in lifespan of AWA ablated animals is modest compared to that of *ocr-2* mutants, suggesting that other *ocr-2*-expressing neurons such as ADL, ASH, ADF and phasmids may play a role in lifespan determination.

Regulators of *C. elegans* insulin secretion are likely to provide insights into mammalian insulin secretion. This was recently highlighted by the demonstration that the mammalian homolog of *asna-1*, encoding an ATPase that functions in *C. elegans* insulin release, is similarly required for insulin secretion from pancreatic β-cells [49]. Emerging experimental evidence also suggest a role for TRPV channels in mammalian insulin secretion (reviewed in [68]). TRPV1 is expressed in neurons that innervate the exocrine and endocrine pancreas [69],[70] as well as insulin-releasing pancreatic β cells [71]. Consistent with our data, treatment of β cell lines by capsaicin, an activator of TRPV1, has been reported to cause a dose-dependent increase in insulin release that is abrogated by simultaneous treatment with capsazepine, a TRPV1 inhibitor [71]. Moreover, various studies have shown that mammalian brain insulin signaling contributes to learning, memory, energy homeostasis, and reproduction (reviewed in [72]). In light of our findings, we speculate that insulin signaling may mediate some of the effects that TRPV1 activation has on neural information storage and adaptation to external cues. Finally, because of their role in pain sensation, there has been intense interest in pharmacological antagonists of TRPV channels. Our findings suggest that such agents could potentially also alter insulin secretion and extend starvation survival and adult lifespan.

## Materials and Methods

### Strain Constructions

Strains were constructed by standard *C. elegans* methods and maintained at either room temperature (∼23°C) or 20°C on NGM (nematode growth media) plates seeded with OP50 *E. coli* as a food source. Dauer constitutive strains were grown and maintained at 15°C. Strains were obtained from the *C. elegans* Genetics Center, CGC (www.cbs.umn.edu/CGC/), unless otherwise noted. Strains KQ35 and KQ170 were generated by backcrossing *unc-31(ft1)* and *unc-31(ft4)*, respectively, 4× with wild type (N2). KQ34 and KQ108 corresponding to *unc-31(ft2)* and *unc-31(ft3)*, respectively, were backcrossed 2× with wild type. KQ190: *tdc-1(ok914)*, KQ191: *tbh-1(ok1196)*, and KQ344: *egl-3(gk238)* were obtained by backcrossing RB993, RB1161, and VC461, respectively, from the OMRF Knockout Consortium (www.mutantfactory.ouhsc.edu/) 4–5× with wild type. MX288: *bbs-1(ok1111)*; *osm-12(n1606)*; *bbs-8 (nx77)* was a gift from Michel Leroux.

Double mutants between *daf-16(mgDf47)* and each of *unc-31(ft1)*, *unc-31(ft4)*, *ocr-2(ak47)*, and *osm-6(p811)* were constructed by mating each single mutant to wild-type males, picking heterozygous males and mating them with hermaphrodites of the second mutant and scoring F2 progeny. *daf-16(mgDf47)* and *ocr-2(ak47)* deletions were scored by PCR. *unc-31(ft1)* and *unc-31(ft4)* were scored by Unc phenotype and by RFLP with RsaI and BstB1, respectively. *osm-6(p811)* was scored by failure to dye-fill with DiO and by RFLP with Hpy188III [73].

KQ1016 was constructed by injection of *Pshr-220::daf-28::mCherry*; *unc-122::GFP*; *Pmyo-2::GFP* into wild type. KQ1041, KQ1042, KQ1044, and KQ1074 were constructed by mating KQ1016 to *ocr-2(ak47)*, *daf-16(mgDf47)*, *unc-31(ft1)*, and *osm-6(p811)* mutants, respectively. KQ1084 was constructed by mating KQ1016 to *ocr-2(ak47)* and selecting for wild-type *ocr-2*. KQ1041, KQ1042, KQ1044, KQ1074 and KQ1084 were used for insulin uptake assay.

### Construction of Transgenic Animals

For cell-specific rescue experiments, *unc-31(ft1)* animals were injected with plasmid of interest at a concentration of 20 ng/µl or 100 ng/µl with *Pmyo-2::GFP* or *Pmyo-3::GFP* at 50 ng/µl as a co-injection marker (see Table S2). Progeny of injected animals were screened for expression of the co-injection marker and mCherry. Stable high-transmitting lines were selected for further studies. *Pshr-220::daf-28::mCherry* was injected at 100 ng/µl along with *unc122::gfp* at 10 ng/µl and co-injection marker *Pmyo-2::GFP* at 50 ng/µl. Transgenic animals were documented using Zeiss Axioplan 2 microscope with Openlab software.

### Plasmid and *unc-31* cDNA Rescue constructs

Plasmids were constructed using Gateway Technology. The *unc-31* cDNA from M_133_ of Wormbase Release WS189 annotated gene structure of *unc-31* to the stop codon was cloned by reverse transcribing cDNA from wild-type mRNA using primers specific to the 5′ and 3′ end of *unc-31* followed by PCR with primers containing attB1 and attB2 sites and cloned into Gateway pDONR-221 vector (Invitrogen). Note that position M_133_ was annotated as the start codon of *unc-31* in Wormbase Release WS160 and the resulting cDNA fully rescued *unc-31(ft1)* mutants. Clones were sequenced and mutations fixed by Stratagene site-directed mutagenesis. For *daf-28*, the corresponding genomic sequence from start to the codon immediately preceding the stop codon was PCR amplified from wild-type genomic DNA and cloned into Gateway pDONR-221 vector and sequenced to verify for accuracy.

Promoters for *egl-3*, *osm-6*, *tax-4*, and *ceh-23* were constructed by the Vidal Lab Promoterome [74] and obtained from Open Biosystems. Promoters for *myo-2* and *myo-3* were gifts from Marc Vidal [74]. Promoters for *shr-220*, *glr-2*, *glr-5*, *glr-8*, *ocr-2*, *gpa-11* and *gpa-13* were constructed by PCR from wild-type genomic DNA using the Vidal Lab Promoterome primers and cloned into pDONR-P4-P1R vector (Invitrogen). Rescue constructs were generated using the pKA460 plasmid to obtain *promoter::unc-31::intercistronic::mCherry* polycistronic fusions. This resulted in the expression of *mCherry* from the same transcript as *unc-31* without modifying *unc-31*. *Posm-6::unc-31* was constructed by cloning the *osm-6* promoter with *unc-31* cDNA into pDEST-MB14, a gift from Marc Vidal [74].

pKA460 was constructed by cutting pDEST-MB14 [74] with SpeI/SacII to remove the *unc-119* rescue fragment and religating the vector to generate pKA296. The GFP sequence was excised from pKA296 with AgeI/EcoRI and replaced with a PCR fragment containing the *mCherry* sequence to generated pKA452. The intercistronic sequence between *gpd-2* and *gpd-3* was PCR amplified from wild-type genomic DNA and cloned into the AgeI site of pKA452. Correct orientation of the insertion was verified by sequencing. *Pmyo-2::GFP* and *Pmyo-3::GFP* were obtained by cloning *myo-2* and *myo-3* promoter into pKA370 destination vector. pKA370 was constructed by digesting pDEST-DD04, a gift from Marc Vidal [74], with NotI to remove the *unc-119* rescue fragment and religating the vector. *Pshr-220::daf-28::mCherry* was constructed by Gateway LR reaction between *Pshr-220*, *daf-28* donor vector and pKA452 destination vector described above.

### L1 Starvation Survival Assay

Worms were grown for at least two generations at either ∼23°C or 20°C in an uncrowded state and treated with hypochlorite to collect embryo [13], which hatched overnight on a rotator in S-basal without cholesterol. Resulting L1 progeny were plated onto 2–3 10 cm NGM plates seeded with OP50, at an uncrowded density and allowed to grow until gravid adults. Embryos were collected once again by bleaching and hatched as described above. Synchronized L1s were then maintained in 125 ml flasks at a concentration of ∼1 worm/µl in 10–12 ml of S-basal without cholesterol supplemented with an antibiotic/antimycotic mix (Invitrogen). Flasks were shaken at 150 rpm on a bench-top shaker at 23°C except when noted. To assess viability, 200 or 300 µl aliquots were taken every 2–3 days and plated onto 6 cm NGM plates seeded with OP50. Plates were monitored using a Zeiss compound light microscope and animals that resumed growth were counted 2–3 days post plating. Survival curves were generated based upon counts of 1000–3000 animals per genotype per experiment. All genotypes that enhanced starvation survival were repeated in multiple independent experiments.

For transgenic rescue experiments, worms were grown at room temperature and synchronized as described above. For each comparison, transgenic and non-transgenic L1s siblings were starved in the same flask as described above. Transgenic and non-transgenic animals were scored based upon the expression of a co-injection marker using a Leica fluorescence microscope. The average from multiple independent transgenic lines for each genotype and their non-transgenic siblings are reported along with the standard error of the mean for each time point.

### Statistical Analysis of Starvation Survival Curves

Log-rank (Mantel-Cox) test was used to analyze starvation survival curves based upon the percentage of survivors at the sampled time points. Statistics were calculated using STATA software. In cases where multiple replicates of a genotype or multiple independent transgenic lines were examined in one experiment, the average survival rate at each time point was determined and that value was used for the reported analyses. To apply log-rank statistics, survival curves were smooth to a non-increasing function to remove noise. For example in Figure 1B, the survival rate of *unc-31(e169)* at day 3 was 90% but at day 6 was 96%, the survival at day 3 was corrected to 96%, as the difference is likely due to an underestimation at day 3. The smoothing only affected the early time points on the survival curves before significant numbers of animals had lost viability. The noise was likely due to pipetting and counting errors and the correction did not significantly change the mean survival rates that were calculated but allowed for application of log-rank statistics. For example, the mean survival rates of *unc-31(e169)* in Figure 1B before and after smoothing were 19.6±0.7 and 19.8±0.6, respectively. The means reported in Table S1 and all survival curves shown are of the raw data before correction.

### Mutagenesis Screen

∼1000 synchronized L4 stage wild-type worms were mutagenized as described in [13] with 47 mM of EMS (ethyl methanesulfonate) along with a mock-treated control. The EMS mutagenized and mock-treated animals were allowed to recover overnight at 20°C and were then bleached to collect ∼15,000 F1 embryos. Resulting F1 animals were plated onto 10 cm NGM plates and incubated until gravid adults at 20°C, which were subsequently bleached to collect synchronized F2s animals. These animals were then starved as described above. After all mock-treated animals were dead, EMS treated F2s were plated onto NGM plates seeded with OP50 and individual animals that could resume growth were picked onto separate plates, allowed to self-fertilize and their progeny retested for enhanced starvation survival. The screen was conducted twice independently and reported results are from the combination of both screens. *ft1* and *ft3* alleles emerged from the first while *ft2* and *ft4* alleles emerged from the second screen.

### Lifespan Assay

Adult lifespan assay and statistical analysis were conducted essentially as previously described [75]. For each genotype, ∼100 L4-stage animals were transferred to NGM plates seeded with OP50 as food source containing 0.1 mg/ml 5-fluor-2′-deoxyuridine (FUDR Sigma), to prevent progeny production. Time of transfer was used as the starting point of the lifespan experiment. Animals that ruptured or crawled off the plate were censored but were included in the analysis up to the time of censor. STATA software was used for statistical analysis as previously described [75]. Note that *ocr-2* mutants used in these lifespan studies were not outcrossed to the Ashrafi-lab wild-type strain. While background mutations can contribute to the lifespan of different “wild-type” strains [76]; the two different alleles of *ocr-2*, *which were* isolated and backcrossed in two independent laboratories, gave very similar results (Figure 6C, Table S3), suggesting that the substantial increase in lifespan of *ocr-2* mutants is unlikely to be caused by background mutations.

### Coelomocyte Insulin Uptake Assay

Strains were synchronized by hypochlorite treatment and the synchronized L1s were plated onto 10 cm NGM plates seeded with OP50. Animals were grown at room temperature for 2 days until L4/young adult stages. Images of the first pair of coelomocytes from 30–50 transgenic animals were recorded at 40× magnification using a Zeiss Axioplan 2 microscope fitted with a Hamamatsu Orca II camera. Images were taken at 15 ms exposure to ensure sub-saturating fluorescence. Fluorescent intensities were quantified using OpenLab software. The outline of each coelomocyte was traced using image from the UNC-122::GFP coelomocyte marker. The fluorescence of DAF-28::mCherry within that area was then measured. The mean fluorescence for each cell was subtracted from the minimum fluorescence (background) within that cell. The mean fluorescence for each coelomocyte along with the standard error of the mean was calculated for each strain and normalized to wild type.

## Supporting Information

Table S1 Log-rank statistical analyses of starvation survival rates.
(0.16 MB DOC)

Table S2 Promoters used for tissue-specific reconstitution of *unc-31*.
(0.07 MB DOC)

Table S3 Log-rank statistical analyses of lifespans.
(0.07 MB DOC)
