# Supplementary material for: A TRPV Channel Modulates C. elegans Neurosecretion, Larval Starvation Survival, and Adult Lifespan
Source: PLoS Genet. 2008 Oct 10;4(10):e1000213. doi: 10.1371/journal.pgen.1000213 (PMC2556084; doi:10.1371/journal.pgen.1000213)
Supplement: Table S1 — Log-rank statistical analyses of starvation survival rates. (0.16 MB DOC) [file pgen.1000213.s001.doc]

**Supplementary Table 1. Log-rank statistical analyses of starvation survival rates**

| Figures | Genotype | Mean  survival^a^ | | p-value  wild type^b^ | p-value  *unc-31*^c^ | p-value  *daf-16*^d^ |
| --- | --- | --- | --- | --- | --- | --- |
| Fig. 1B | wild type | 12.3±0.4^3^ | |  |  |  |
|  | *unc-31(ft2)*^*^ | 17.5±0.8^3^ | | <0.00001 |  |  |
|  | *unc-31(ft3)*^*^ | 17.2±0.7^3^ | | <0.00001 | 0.2568 |  |
|  | *unc-31(ft4)* ^*^ | 18.7±0.8^3^ | | <0.00001 | 0.2231 |  |
|  | *unc-31(e169)* | 19.6±0.7^3^ | | <0.00001 | 0.3485 |  |
|  | *unc-31(e928)* | 17.9±0.7^3^ | | <0.00001 | 0.3566 |  |
|  |  |  | |  |  |  |
| Fig. 2A, 2B^**^ | wild type | 16.8±0.7^2^ | |  |  |  |
|  | *unc-34(e566)* | 18.9±0.6^2^ | | 0.1693 |  |  |
|  | *unc-36(e251)* | 19.4±0.5^2^ | | 0.1590 |  |  |
|  | *unc-60(e723)* | 14.8±0.4^2^ | | <0.00001 |  |  |
|  | *unc-87(e1216)* | 15.3±0.5^2^ | | 0.0001 |  |  |
|  | *unc-31(ft1)* ^*^ | 32.2±0.9^1^ | | <0.00001 |  |  |
|  | *unc-31(e928)* | 31.7±1.2^1^ | | <0.00001 | 0.2194 |  |
|  | *unc-10(e102)* | 20.2±0.7^2^ | | 0.0009 |  |  |
|  | *unc-18(e81)* | 12.0±0.5^2^ | | <0.00001 |  |  |
|  | *unc-64(e246)* | 20.2±0.7^2^ | | 0.0021 |  |  |
|  | *snb-1(md247)* | 18.9±0.7^2^ | | 0.0574 |  |  |
|  | *snt-1(ad596)* | 14.9±0.5^2^ | | 0.0001 |  |  |
|  |  |  | |  |  |  |
| Fig. 2C | wild type | 10.7±0.5^3^ | |  |  |  |
|  | *tdc-1(ok914)* ^*^ | 11.6±0.4^3^ | | 0.4379 |  |  |
|  | *tbh-1(ok1196)* ^*^ | 11.8±0.5^3^ | | 0.0682 |  |  |
|  | *tph-1(mg280)* | 12.4±0.5^1^ | | 0.0059 |  |  |
|  | *cat-2(e1112)* | 10.7±0.4^3^ | | 0.1852 |  |  |
|  |  |  | |  |  |  |
| Fig. 2D | wild type | 12.0±.4 | |  |  |  |
|  | *eat-4(ad819)* | 11.0±.4 | | 0.0402 |  |  |
|  | *eat-4(ky5)* | 11.6±.4 | | 0.2334 |  |  |
|  |  |  | |  |  |  |
| Fig. 2E | wild type | 10.7±0.5^3^ | |  |  |  |
|  | *egl-3(n150)* | 10.7±0.5^3^ | | 0.9678 |  |  |
|  | *egl-3(gk238)* ^*^ | 11.7±0.5^3^ | | 0.1069 |  |  |
|  | *egl-21(n611)* | 9.7±0.4^3^ | | 0.0570 |  |  |
|  |  |  | |  |  |  |
| Fig. 2F | wild type | 12.5±0.3 | |  |  |  |
|  | *unc-31(ft1)* ^*^ | 23.6±0.7 | | <0.00001 |  |  |
|  | *unc-31(ft4)* ^*^ | 26.3±1.2 | | <0.00001 |  |  |
|  | *daf-16(mgDf47)* ^*^ | | 7.5±0.3 | <0.00001 |  |  |
|  | *daf-16(mgDf47);*  *unc-31(ft1)* ^*^ | | 7.6±0.2 | <0.00001 | <0.00001 | 0.8689 |
|  | *daf-16(mgDf47);*  *unc-31(ft4)* ^*^ | | 7.7±0.3 | <0.00001 | <0.00001 | 0.1951 |
|  |  | |  |  |  |  |
| Fig. 3A | wild type | | 13.8±0.5 |  |  |  |
|  | *daf-1(m40)* | | 14.8±0.5 | 0.0577 |  |  |
|  | *daf-3(mgDf90)* | | 13.7±0.5 | 0.6348 |  |  |
|  | *daf-7(e1372)* | | 13.1±0.6 | 0.4938 |  |  |
|  | *daf-7(m62)* | | 13.8±0.5 | 0.7746 |  |  |
|  | *daf-12(m20)* | | 13.6±0.5 | 0.5726 |  |  |
|  | *unc-31(ft1)* ^*^ | | 21.0±0.8 | <0.00001 |  |  |
|  | *daf-16(mgDf47)* ^*^ | | 5.8±0.2 | <0.00001 |  |  |
|  |  | |  |  |  |  |
| Fig. 3B | wild type | | 12.0±0.4 |  |  |  |
|  | *eat-2(ad465)* | | 8.3±0.3 | <0.00001 |  |  |
|  | *eat-2(ad1116)* | | 10.0±0.3 | <0.00001 |  |  |
|  | *unc-31(ft1)* ^*^ | | 19.4±0.9 | <0.00001 |  |  |
|  | *daf-16(mgDf47)* ^*^ | | 5.4±0.2 | <0.00001 |  |  |
|  |  | |  |  |  |  |
| Fig. 3C^**^ | wild type | | 16.8±0.6 |  |  |  |
|  | *clk-1(e2519)* | | 13.7±0.6 | <0.00001 |  |  |
|  | *clk-1(qm30)* | | 12.1±0.4 | <0.00001 |  |  |
|  | *isp-1(qm150)* | | 11.9±0.3 | <0.00001 |  |  |
|  |  | |  |  |  |  |
| Fig. 5A | wild type | | 10.4±0.3 |  |  |  |
|  | *osm-1(p808)* | | 16.4±0.5 | <0.00001 | 0.3660 |  |
|  | *osm-6(p811)* | | 18.3±0.4 | <0.00001 | 0.0521 |  |
|  | *unc-31(ft1)* ^*^ | | 19.1±0.6 | <0.00001 |  |  |
|  | *bbs-1(ok1111); osm-12(n1606);*  *bbs-8(nx77)* | | 10.6±0.3 | 0.1234 |  |  |
|  |  | |  |  |  |  |
| Fig. 5B | wild type | | 8.5±0.1 |  |  |  |
|  | *che-2(e1033)* | | 8.5±0.2 | 0.4297 |  |  |
|  | *che-3(e1124))* | | 8.6±0.3 | 0.9824 |  |  |
|  | *che-11(e1810)* | | 12.2±0.4 | <0.00001 |  |  |
|  | *osm-3(p802)* | | 13.0±0.5 | <0.00001 |  |  |
|  |  | |  |  |  |  |
| Fig. 5C | wild type | | 10.8±0.4^3^ |  |  |  |
|  | *osm-6(p811)* | | 14.8±0.6^3^ | <0.00001 |  |  |
|  | *daf-16(mgDf47)* ^*^ | | 4.6±0.2^3^ | <0.00001 |  |  |
|  | *daf-16(mgDf47); osm-6(p811)* ^*^ | | 4.3±0.1^3^ | <0.00001 |  | 0.1427 |
|  |  | |  |  |  |  |
| Fig. 5D, 5E | wild type | | 9.6±0.3^3^ |  |  |  |
|  | *ocr-1(ak46)* | | 8.4±0.4^3^ | 0.1117 |  |  |
|  | *ocr-2(ak47)* | | 12.4±0.6^1^ | <0.00001 | 0.0176 |  |
|  | *ocr-2(yz5)* | | 12.6±0.6^3^ | <0.00001 | 0.0136 |  |
|  | *ocr-4(vs137)* | | 9.3±0.4^3^ | 0.6492 |  |  |
|  | *osm-9(ky10)* | | 9.9±0.4^3^ | 0.0440 |  |  |
|  | *unc-31(ft1)* ^*^ | | 14.6±0.6^3^ | <0.00001 |  |  |
|  | *daf-16(mgDf47)* ^*^ | | 4.7±0.2^2^ | <0.00001 |  |  |
|  | *daf-16(mgDf47);*  *ocr-2(ak47)* ^*^ | | 4.9±0.2^3^ | <0.00001 |  | 0.3005 |
|  |  | |  |  |  |  |
| Fig. 5F | wild type | | 10.0±0.3 |  |  |  |
|  | *odr-3(n2150)* | | 8.0±0.3 | <0.00001 |  | <0.00001 |
|  | *unc-31(ft1)* ^*^ | | 16.1±0.5 | <0.00001 |  |  |
|  | *daf-16(mgDf47)* ^*^ | | 4.9±0.2 | <0.00001 |  |  |

Mean survival and statistical significance were calculated for each experiment as detailed in Materials and Methods. Strains denoted by ^*^ were outcrossed to out laboratory’s wild-type strain as detailed in Materials in Methods. Experiments were carried out at room temperature (~23°C) unless otherwise noted by ^**^ which were conducted at 20°C.

^a^ Mean survival and standard error for indicated genotypes. In cases where multiple replicates of a given genotype were tested in an experiment, the reported mean is based on the average survival curve of the replicates. Superscript indicates the number of replicates of that genotype in that experiment.

^b^ p-values relative to wild type strain in that experiment by log-rank test.

^c^ p-values relative to *unc-31* mutant in that experiment by log-rank test.

^d^ p-values relative to *daf-16(mgDf47)* in that experiment by log-rank test.
