# Supplementary material for: A TRPV Channel Modulates C. elegans Neurosecretion, Larval Starvation Survival, and Adult Lifespan
Source: PLoS Genet. 2008 Oct 10;4(10):e1000213. doi: 10.1371/journal.pgen.1000213 (PMC2556084; doi:10.1371/journal.pgen.1000213)
Supplement: Table S2 — Promoters used for tissue-specific reconstitution of unc-31. (0.07 MB DOC) [file pgen.1000213.s002.doc]

**Supplementary Table 2. Promoters used for tissue-specific reconstitution of *unc-31***

| Promoter | Promoter length ^a^ | Expression pattern | Conc.  injected | Rescue *unc-31(ft1)* ^b^ | p-value  +/- ^c^ |
| --- | --- | --- | --- | --- | --- |
| *egl-3*^d^ | 2000bp | Pan-neuronal [35] | 20ng/ul | full | <0.00001 |
| *myo-3* | 2500bp | Body wall muscle [36] | 20ng/ul | none | 0.7134 |
| *myo-2* | 2509bp | Pharyngeal muscle [36] | 20ng/ul | none | 0.9612 |
| *osm-6*^d^ | 427bp | Ciliated neurons [37] | 20ng/ul | full | <0.00001 |
| *glr-5* | 1609bp | Many interneurons [38] | 100ng/ul | none | 0.1233 |
| *glr-2* | 1638bp | 24 interneurons [38] | 20ng/ul | none | 0.9630 |
| *glr-8^e^* | 2000bp | 13 pharyngeal neurons and 5 interneurons [38] | 20ng/ul | none | 0.0041^e^ |
| *ceh-23*^d^ | 2000bp | **ADL, ASH**, AWC, ASI, AFD, ASG, BAG, ASE, **phasmids**, CAN, AIY [39] | 100ng/ul | full | <0.00001 |
| *tax-4*^d^ | 2001bp | AWC, ASI, AFD, ASG, ASJ, ASK, BAG, URX, ASE [40] | 100ng/ul | partial | <0.00001 |
| *ocr-2*^d^ | 2000bp | ADL, ASH, AWA, ADF, phasmids [41] | 100ng/ul | partial | 0.0004 |
| *gpa-11*^d^ | 2000bp | ADL, ASH [42] | 20ng/ul  100ng/ul | partial  partial | 0.0002  0.0007 |
| *gpa-13* | 2000bp | AWC, ADF, ASH, phasmids [42] | 20ng/ul | none | 0.4254 |

^a^ Promoter lengths are calculated from the start codon of corresponding genes. Ciliated neurons targeted by the *ceh-23* but not by the *tax-4* promoter are shown in bold.

^b^ Rescue was defined as reversion of extended starvation survival of *unc-31* mutants to wild-type levels as determined by log-rank test. Mean survival rates of transgenic animals that were statistically different from both non-trangenic siblings and wild type were considered partial rescues.

^c^ p-values were calculated between transgenic (+) and non-transgenic (-) siblings in that experiment by log-rank test.

^d^ Promoters used to express wild-type *unc-31* cDNA that abrogated extended survival of *unc-31(ft1)*.

^e^ Neither transgenic nor non-transgenic animals were statistically different from *unc-31 (ft1)*.
