# Supplementary material for: A TRPV Channel Modulates C. elegans Neurosecretion, Larval Starvation Survival, and Adult Lifespan
Source: PLoS Genet. 2008 Oct 10;4(10):e1000213. doi: 10.1371/journal.pgen.1000213 (PMC2556084; doi:10.1371/journal.pgen.1000213)
Supplement: Table S3 — Log-rank statistical analyses of lifespans. (0.07 MB DOC) [file pgen.1000213.s003.doc]

**Supplementary Table 3. Log-rank statistical analyses of lifespans**

| Genotype | Mean  lifespan | % wild type^a^ | Number of  animals^b^ | p-value  wild type^c^ | p-value  *ocr-2*^d^ | p-value  *daf-16*^e^ |
| --- | --- | --- | --- | --- | --- | --- |
| Experiment 1 |  |  |  |  |  |  |
| wild type | 17.2 |  | 80/95 |  |  |  |
| *ocr-2(ak47)* | 26.6 | 155% | 87/97 | <0.00001 |  |  |
| *unc-31(ft1)* | 33.9 | 197% | 94/95 | <0.00001 | <0.00001 |  |
|  |  |  |  |  |  |  |
| Experiment 2 |  |  |  |  |  |  |
| wild type | 19.3 |  | 55/86 |  |  |  |
| *ocr-2(ak47)* | 31.2 | 162% | 74/82 | <0.00001 |  |  |
| *daf-16(mgDf47)* | 14.0 | 72% | 41/83 | <0.00001 |  |  |
| *daf-16(mgDf47);*  *ocr-2(ak47)* | 13.7 | 71% | 32/57 | <0.00001 | <0.00001 | 0.6858 |
|  |  |  |  |  |  |  |
| Experiment 3* |  |  |  |  |  |  |
| wild type | 19.2 |  | 64/97 |  |  |  |
| *ocr-2(ak47)* | 29.8 | 155% | 79/92 | <0.00001 |  |  |
| *ocr-2(yz5)* | 27.0 | 141% | 31/62 | <0.00001 | 0.0819 |  |
| *unc-31(ft1)* | 33.6 | 175% | 90/98 | <0.00001 | 0.0033 |  |
| *daf-16(mgDf47)* | 14.1 | 73% | 56/97 | <0.00001 |  |  |
| *daf-16(mgDf47);*  *ocr-2(ak47)* | 14.8 | 77% | 37/66 | <0.00001 | <0.00001 | 0.1012 |

Lifespans were determined on plates at 20°C with 0.1mg/ml FUDR to inhibit progeny production. Note that the *ocr-2* mutants had not been outcrossed to the laboratory wild- type strains; however, both independently derived alleles of *ocr-2*, *ak47* and *yz5*, gave very similar results and both substantially increased lifespan compared to the laboratory wild-type strain.

* Denotes experiment shown in Figure 6C.

^a^ Lifespan relative to that of wild type in that experiment.

^b^ Number of (observed dead animals)/(total animals at start of experiment). The difference is due to censoring of animals that crawled off the plate or ruptured.

^c^ p-values relative to wild type by log-rank test.

^d^ p-values relative to *ocr-2(ak47)* by log-rank test.

^e^ p-values relative to *daf-16(mgDf47)* by log-rank test.
